# Turpentine in Typhoid Fever

**Published:** 1892-11

**Authors:** 


### TURPENTINE IN TYPHOID FEVER.

H. C. Wood believes that when, in the convalescence of typhoid fever, the existence of local intestinal symptoms points toward slowness of healing of the ulcers, turpentine is an invaluable remedy. It is also indicated when there is marked typanites, with dryness of the tongue, developing in the end of the second week of typhoid fever. The action of turpentine is believed to be a local one upon the ulcerated surface. The terebinthinates are slowly absorbed, and, indeed, volatilized at the temperature of the stomach and intestines, and we readily believe, from the result of the laboratory researches, that there is a special relation between the oil of turpentine and the bacillus of typhoid fever (Omelchenko). This practice has been in vogue for more than half a century, and the author believes that it is a good one, and distinctly tends to lessen the severity of the local lesions in enteric fever. The formula recommended is oil of turpentine, 1; glycerin, 4; mucilage of acacia, 6; peppermint water to 32. The dose is one teaspoonful every four hours during the day.—*Therapeutic Gazette*, 1892, No. 6, p. 366.

[This sound practice, based upon intelligent empiricism of half a century ago, has recently received support from the results of laboratory experiments, and is now placed upon a scientific basis; and it is likely to outlast many of the modern methods now so much in vogue.—R. W. W.)—*American Jour. Med. Science*, Oct. 1892.

